# Supplementary material for: Integrative analysis of RNA binding proteins identifies DDX55 as a novel regulator of 3’UTR isoform diversity
Source: Genome Biol. 2025 Nov 12;26:386. doi: 10.1186/s13059-025-03852-8 (PMC12606947; doi:10.1186/s13059-025-03852-8)
Supplement: Supplementary file 3 — Additional file 3: Figures S1-S17. [file 13059_2025_3852_MOESM3_ESM.pdf]

SUPPLEMENTAL INFORMATION

Integrative analysis of RNA binding proteins identifies DDX55 as a novel regulator of 3'UTR isoform diversity

Matthew R. Gazzara[1,2,3], Timothy Cater[1,4], Michael J. Mallory[1], Yoseph Barash[2,3,4,5,#], Kristen W. Lynch[1,3,4,#]

[1]Department of Biochemistry and Biophysics, [2]Genomics and Computational Biology Graduate Group, [3]Department of Genetics, and [4]Genetics and Epigenetics Graduate Group

Perelman School of Medicine and [5]Department of Computer and Information Science, School of Engineering and Applied Science, University of Pennsylvania, Philadelphia, PA, 19104, USA

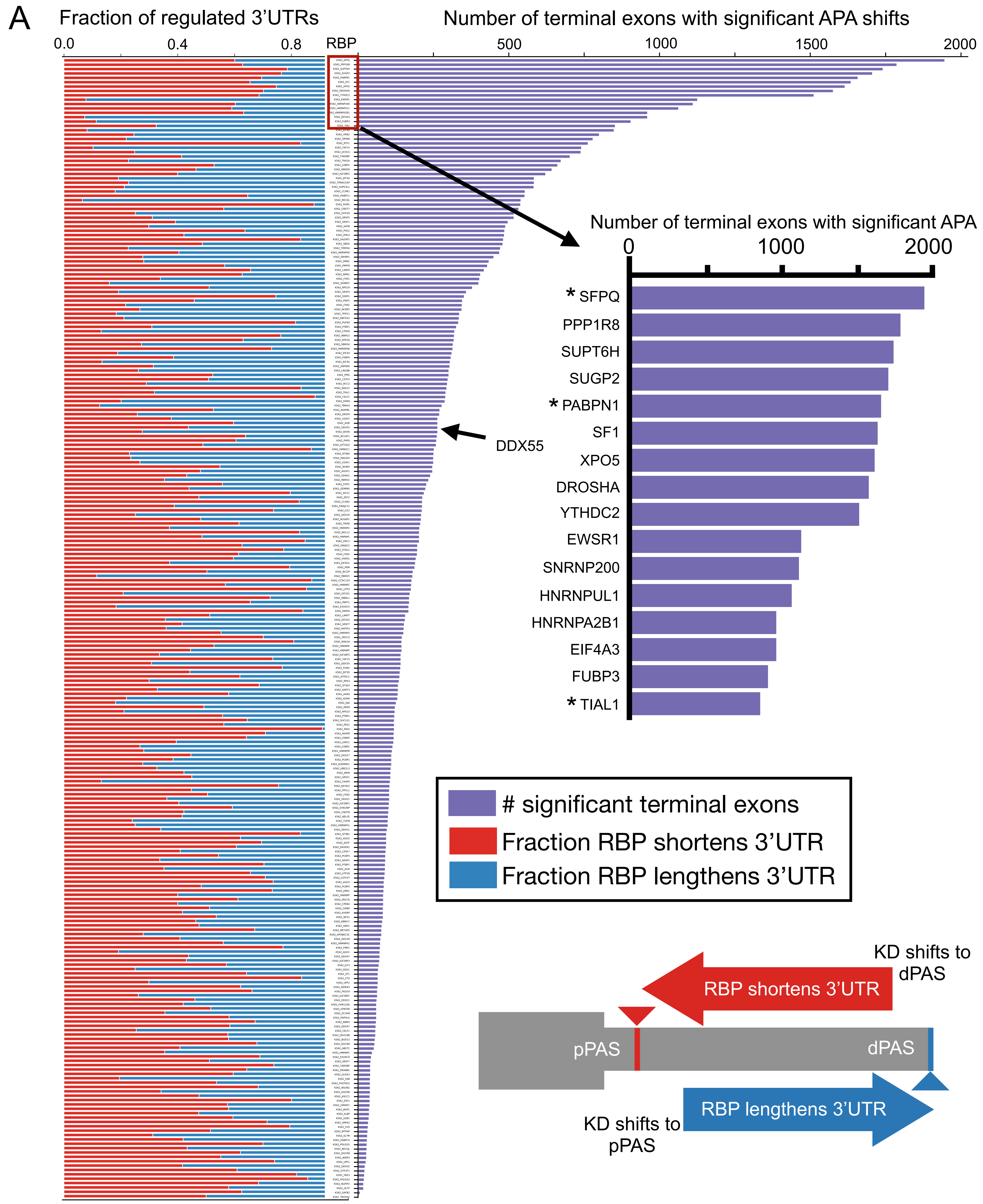

Fig. S1: **The landscape of 3'UTR tandem APA regulation in K562 cells**

(A) Summary of DaPars analysis of all K562 RBP knockdowns from ENCODE (244 shRNA mediated RBP depletion experiments) showing the number of significant terminal exons (right, purple, |dDPUI|$\geq$20% with adjusted $p<0.05$). Right inset shows the top 16 RBPs by number of genes regulated. Stacked bar chart indicates the fraction of significant genes that had a pattern of RBP shortens 3'UTR (red, left bars) or RBP lengthens 3'UTR (blue, right bars) as described the inset cartoon. All experiments analyzed and counts are listed in Supplementary Tables 1 and 2.

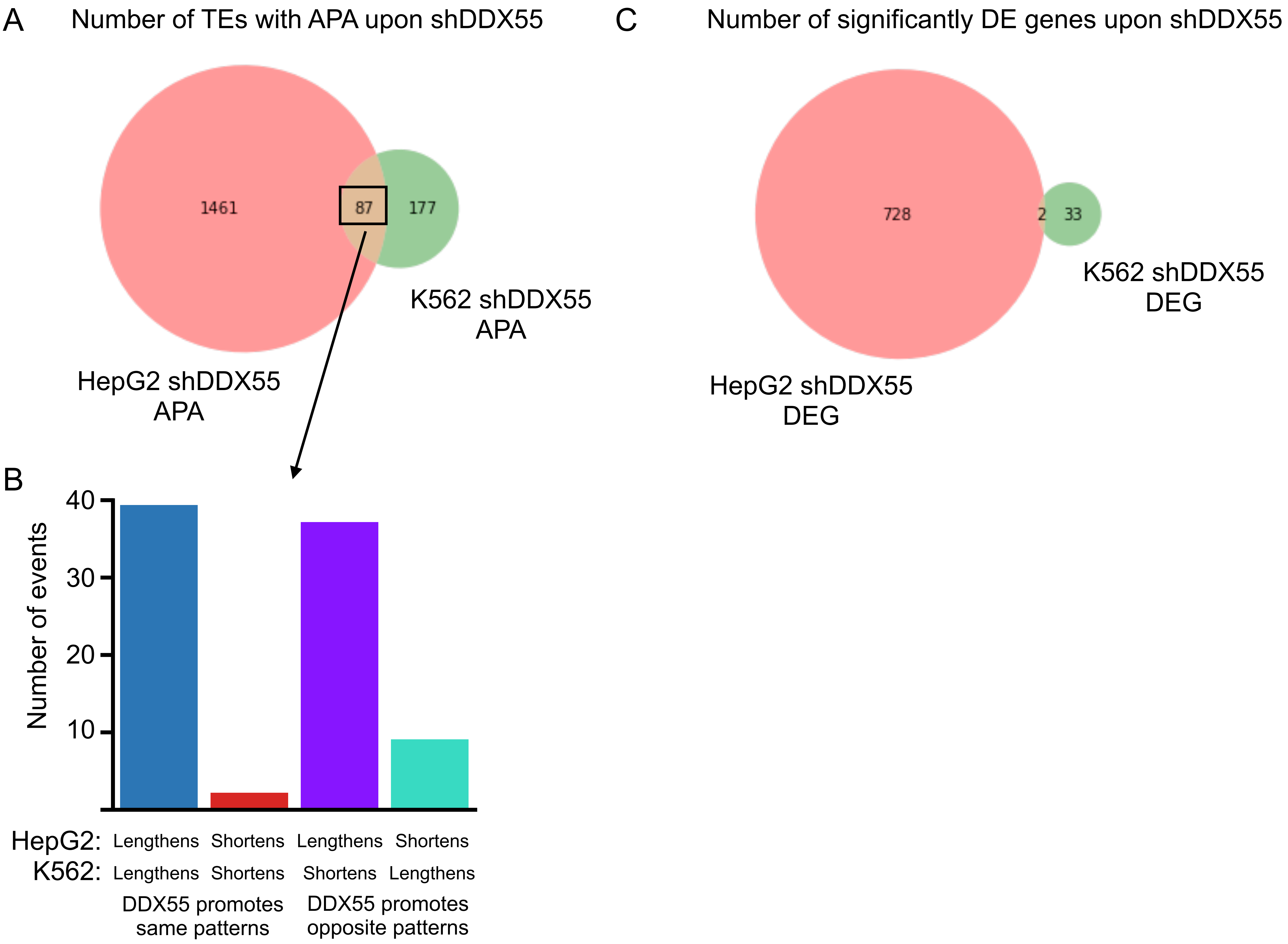

Fig. S2: **Shared APA shifts across cell types**

(A) Venn diagram displaying number of terminal exons regulated by DDX55 in HepG2 cells (red) or K562 cells (green) **(B)** Counts of terminal exons that are regulated by DDX55 in both HepG2 and K562 depletion experiments by event classes. **(C)** Overlap of DESeq2 called significantly differentially expressed genes (log2(FC)$\geq$2 and *p*-adjusted < 0.05) for DDX55 depletion in HepG2 cells (red) or K562 cells (green).

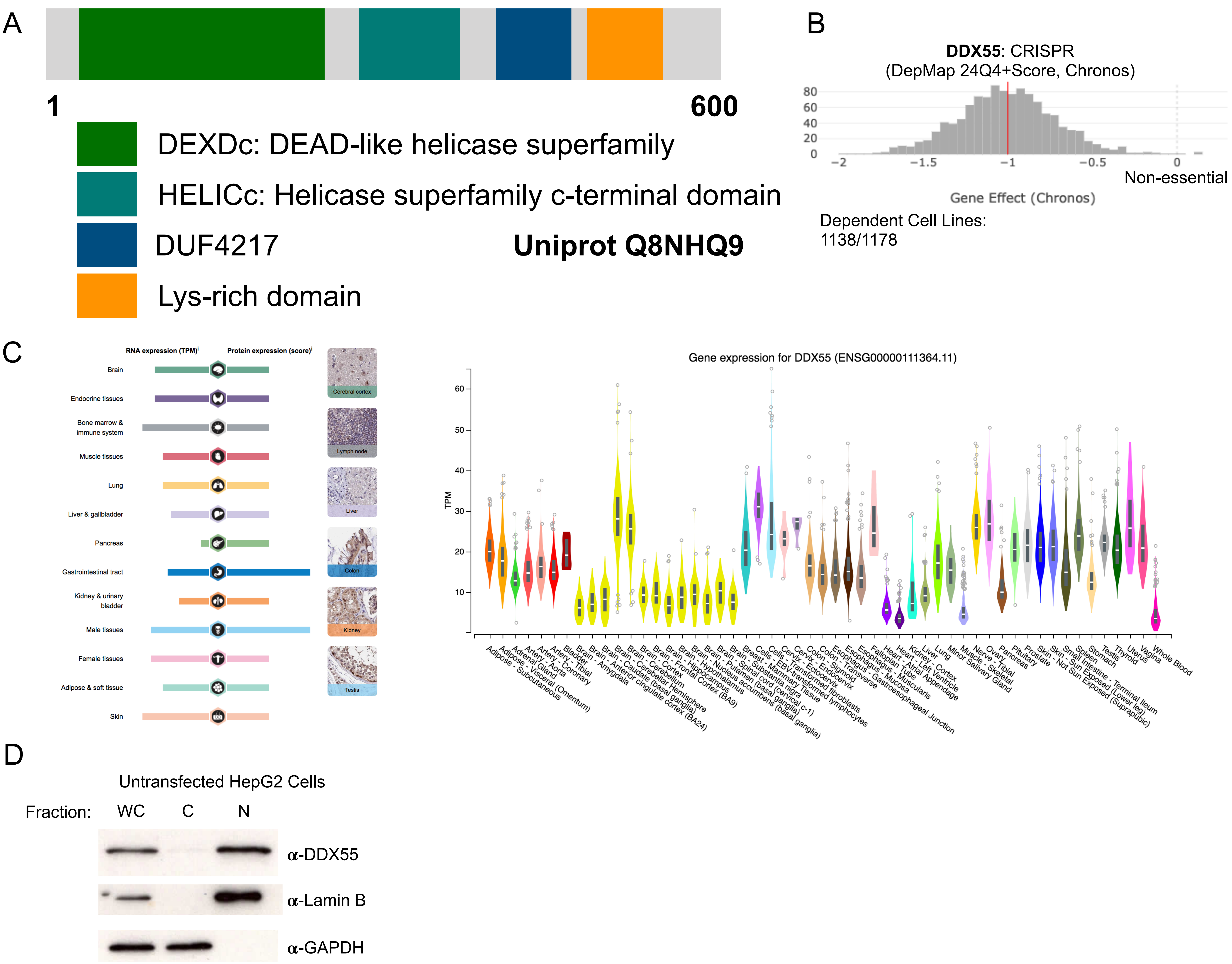

Fig. S3: **Characteristics and expression pattern of DDX55**

(**A**) Domain structure of DDX55 from Uniprot. (**B**) DepMap essential gene results showing gene effect prediction from CRISPR knockout in cell lines. Red line and score of -1 indicates the median value observed for pan-essential genes and 0 indicates non-essential. 1,138 out of 1,178 cell lines were found to be dependent on DDX55. (**C**) Human Protein Atlas summarizing RNA and protein expression of DDX55 across different tissues (left) and GTEx steady state mRNA expression level of DDX55 across indicated tissues. (**D**) Western blot on protein lysates from whole cell (WC), cytoplasmic (C), and nuclear (N) fractions from wildtype, untreated HepG2 cells. GAPDH and Lamin B served as controls for cytoplasmic and nuclear fractionation, respectively.

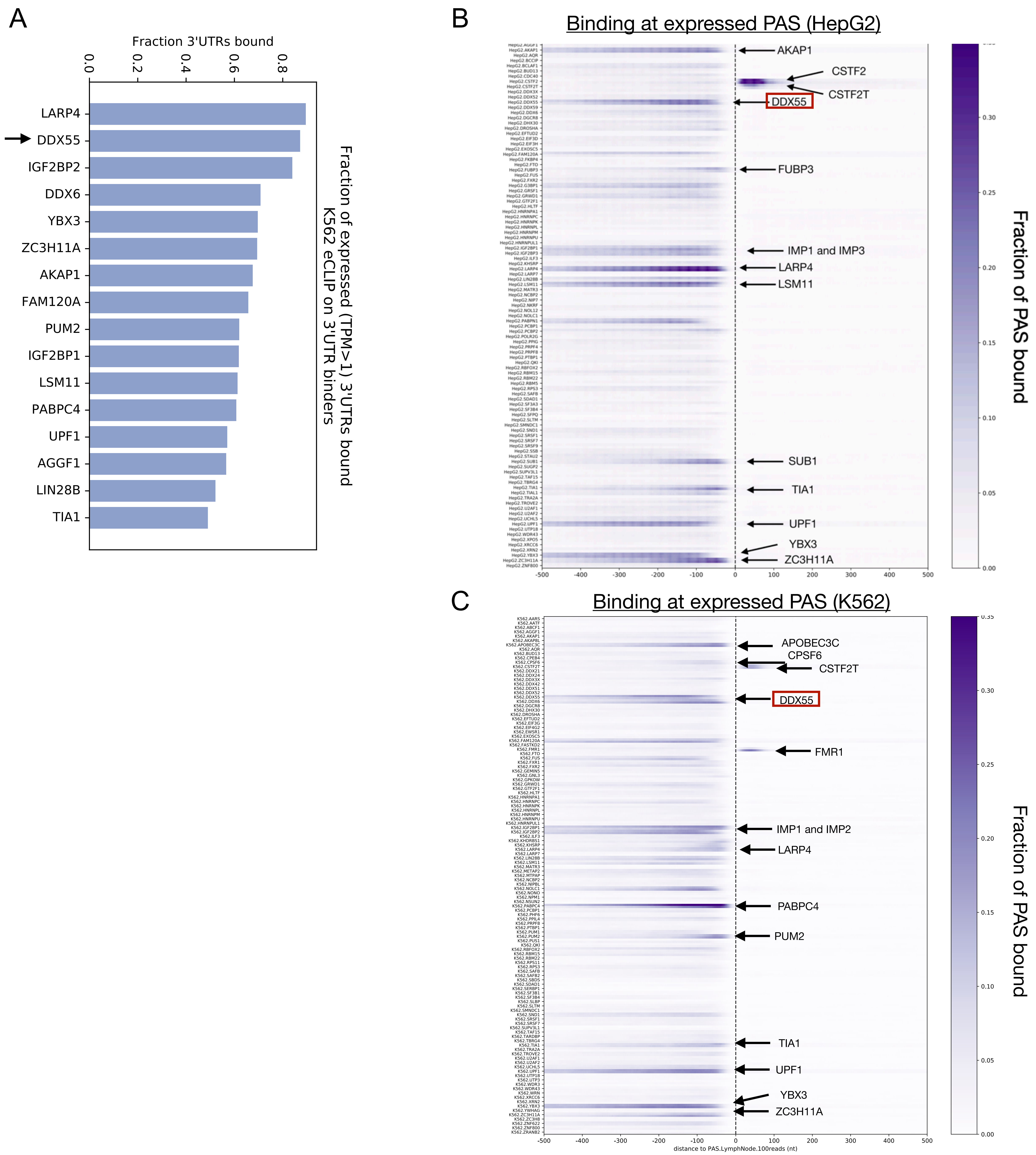

Fig. S4: **RBP eCLIP around 3' ends in K562 and HepG2 cells after extended peak calls**

(**A**) Fraction of expressed mRNA transcripts (TPM≥1) from K562 cells that had evidence of RBP binding in K562 cells (presence of any eCLIP peak) within the 3'UTR. (**B**) Heatmap showing the fraction of highly expressed PAS from human liver (100+ reads) that had evidence of proximal eCLIP binding (after downstream addition to peak calls) for all RBPs in HepG2 cells on a per-nucleotide basis within 500 nt up or downstream of the PAS. Select RBPs of interest are highlighted. (**C**) Same as panel (B) but for K562 cells. Select RBPs of interest are highlighted.

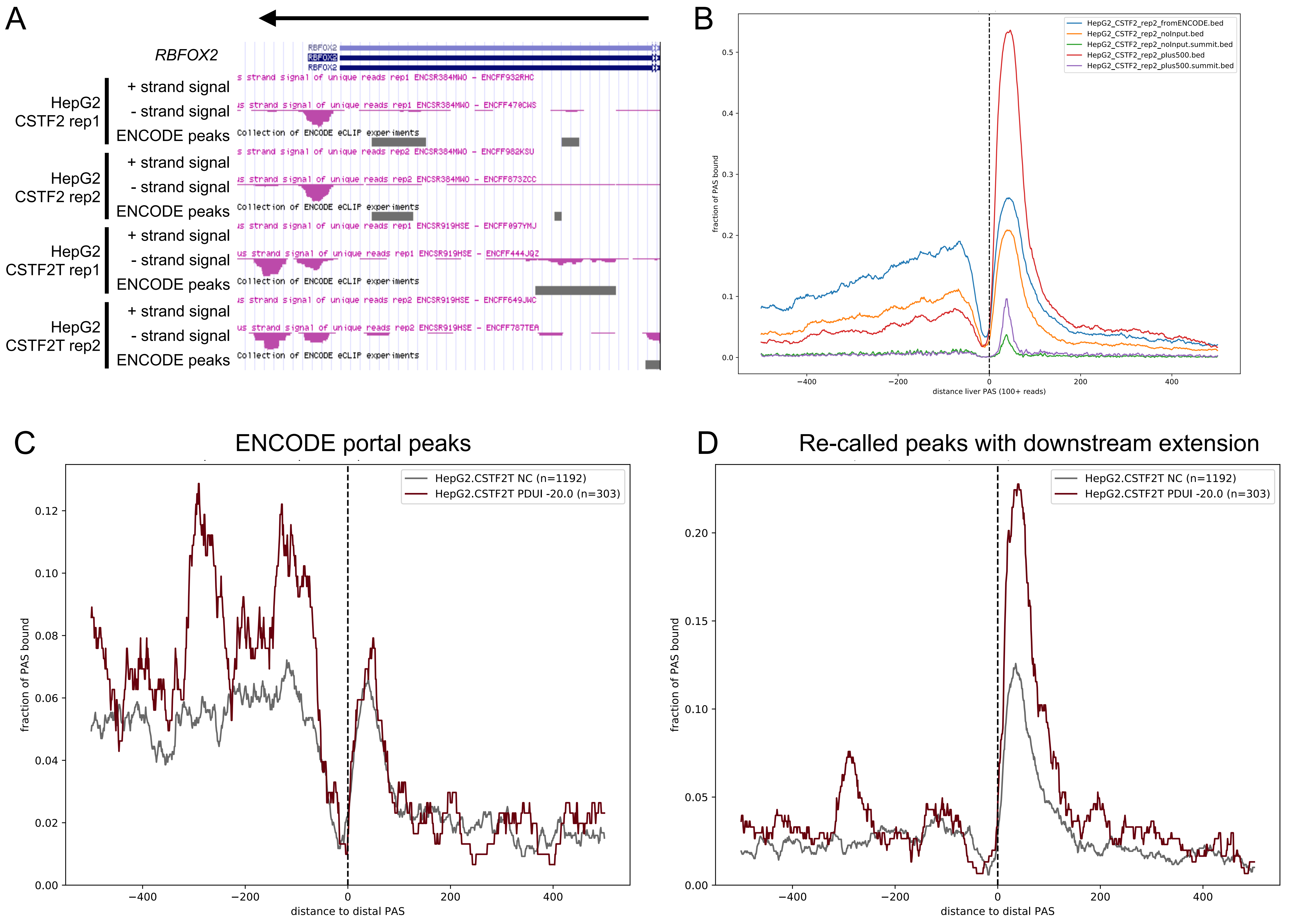

Fig. S5: Correcting ENCODE eCLIP peak calling on RBPs that bind downstream of the terminal PAS

(**A**) UCSC genome browser view of minus strand gene *RBFOX2*'s 3'end with ENCODE portal tracks for CSTF2 (CstF64) and CSTF2T (CstF64t) eCLIP read coverage from HepG2 cells (fuchsia). ENCODE called eCLIP peaks are shown in gray. (**B**) RNA map meta plot centered on highly expressed PAS from human liver (100+ reads) showing the fraction of PAS with HepG2 CSTF2 eCLIP peaks called by various methods labeled in the inset. (**C**) RNA map meta-transcript plot centered on the distal PAS showing the fraction of events with HepG2 CSTF2T eCLIP peaks called by ENCODE at each position around non-changing genes (gray) and genes where CSTF2T normally promotes transcript shortening (red). (**D**) Same as (C), but for HepG2 CSTF2T peaks re-called with a downstream extension of 500 nt to annotated transcript end.

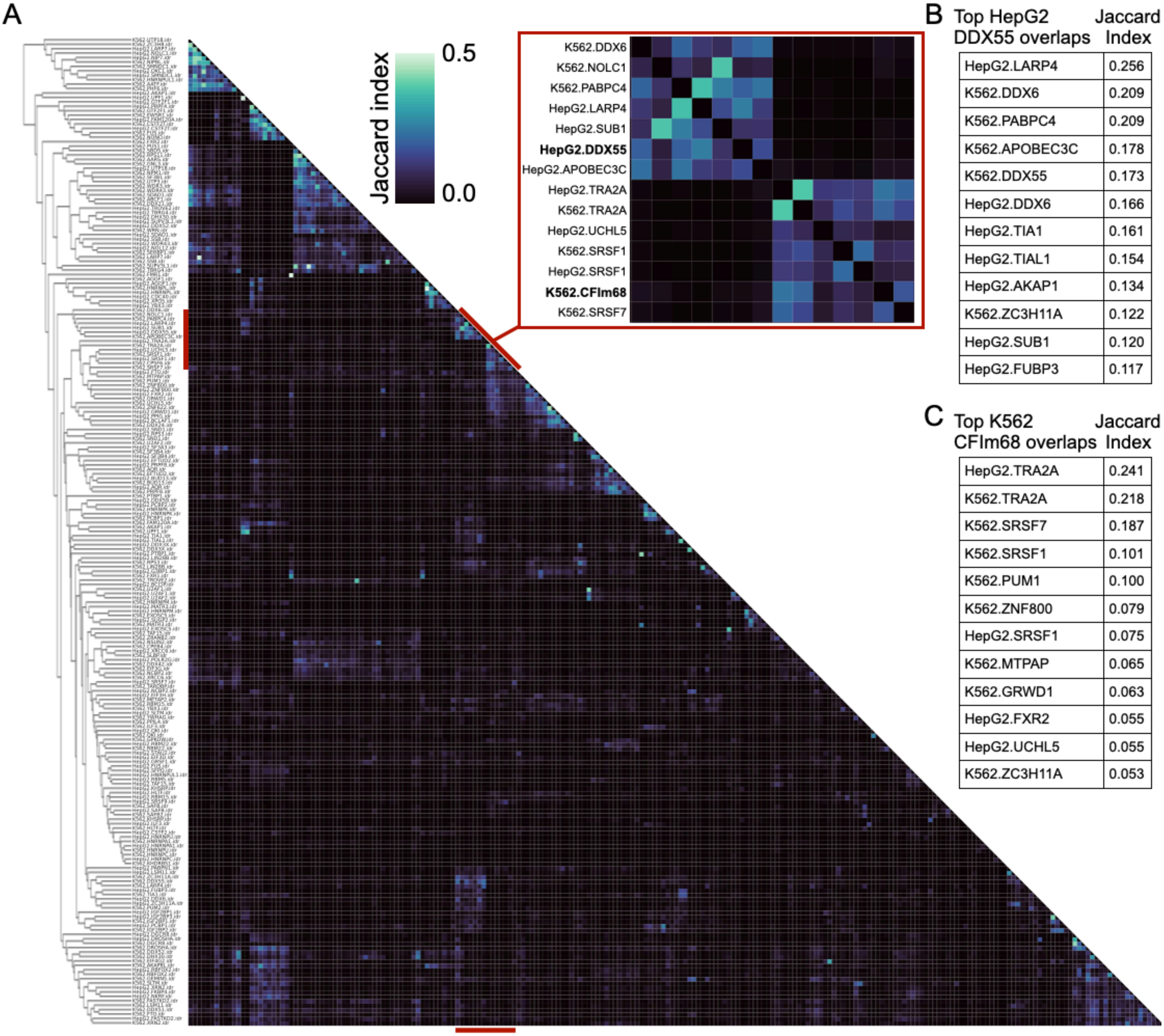

B

Top HepG2 DDX55 overlaps	Jaccard Index
HepG2.LARP4	0.256
K562.DDX6	0.209
K562.PABPC4	0.209
K562.APOBEC3C	0.178
K562.DDX55	0.173
HepG2.DDX6	0.166
HepG2.TIA1	0.161
HepG2.TIAL1	0.154
HepG2.AKAP1	0.134
K562.ZC3H11A	0.122
HepG2.SUB1	0.120
HepG2.FUBP3	0.117

C

Top K562 CFIm68 overlaps	Jaccard Index
HepG2.TRA2A	0.241
K562.TRA2A	0.218
K562.SRSF7	0.187
K562.SRSF1	0.101
K562.PUM1	0.100
K562.ZNF800	0.079
HepG2.SRSF1	0.075
K562.MTPAP	0.065
K562.GRWD1	0.063
HepG2.FXR2	0.055
HepG2.UCHL5	0.055
K562.ZC3H11A	0.053

Fig. S6: Pairwise eCLIP overlaps

(**A**) Heatmap showing hierarchical clustering of Jaccard Index for all pairwise IDR eCLIP peak sets. Inset highlights two clusters that contain DDX55 and the CFIm subunit CPSF6 (CFIm68). (**B**) Top 12 IDR peaks from eCLIP experiments indicated that overlap with HepG2 DDX55 IDR peaks, sorted by Jaccard index. (**C**) As in (B), but for overlaps with K562 CPSF6 (CFIm68) IDR peaks

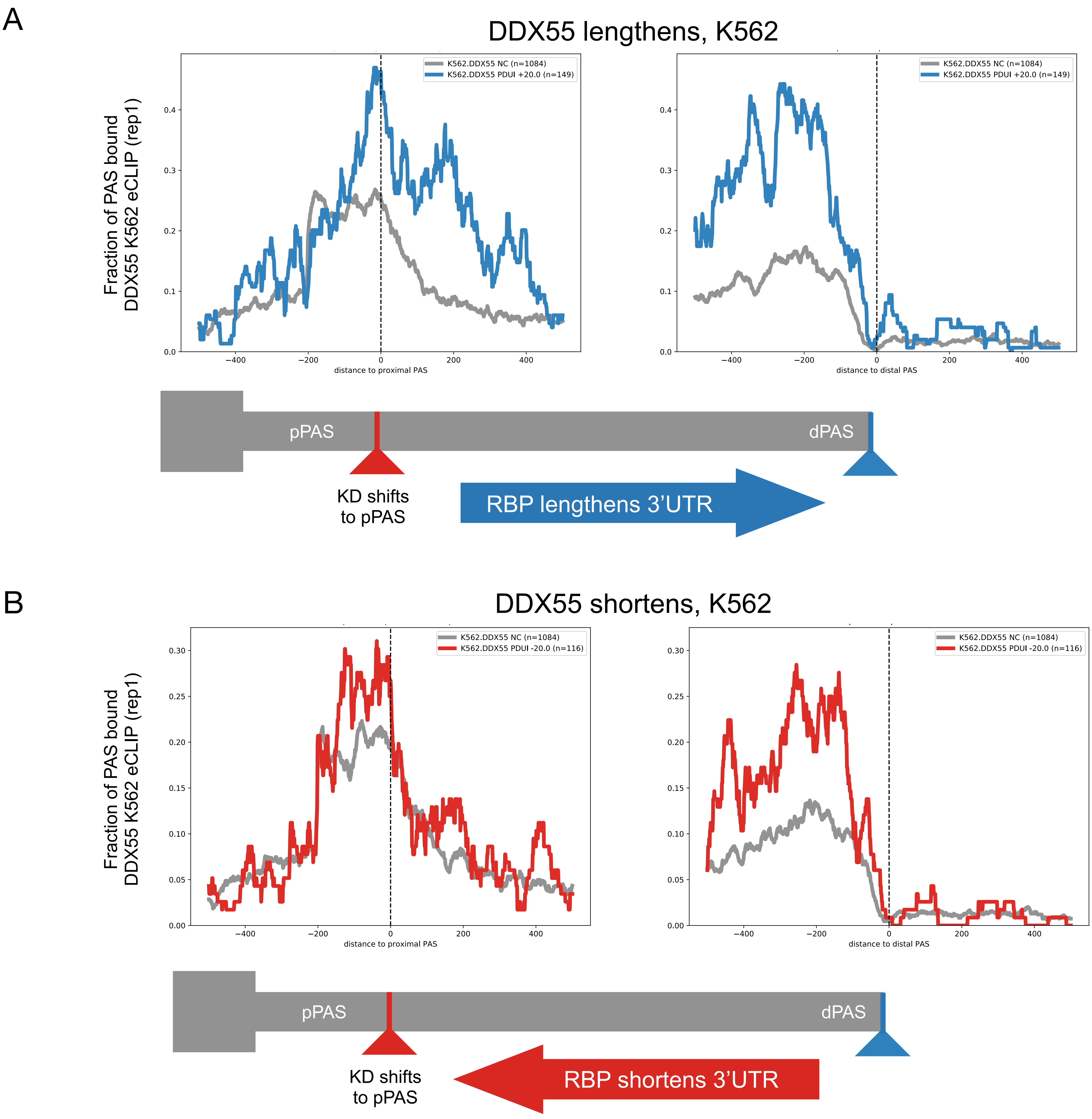

Fig. S7: **RNAmaps DDX55 tandem 3’UTR regulation in K562 cells**

(**A**) RNA map of the fraction of events with K562 DDX55 eCLIP peaks (replicate 1 shown) centered around proximal polyadenylation sites (pPAS, left) or distal PAS (dPAS, right) for events in K562 cells where DDX55 promotes 3’UTR long isoform expression (blue line, knockdown (KD) shifts expression towards pPAS, dDPUI≥20% with FDR<0.05) or events were DDX55 depletion had no effect on PAS choice (gray line, |dPDUI|≤5% with FDR>0.05). (**B**) same as panel (A) but for events in K562 cells where DDX55 promotes 3’UTR short isoform expression (red line, knockdown (KD) shifts expression towards dPAS, dDPUI≤-20% with FDR<0.05) or events were DDX55 depletion had no effect on PAS choice (gray line, |dPDUI|≤5% with FDR>0.05).

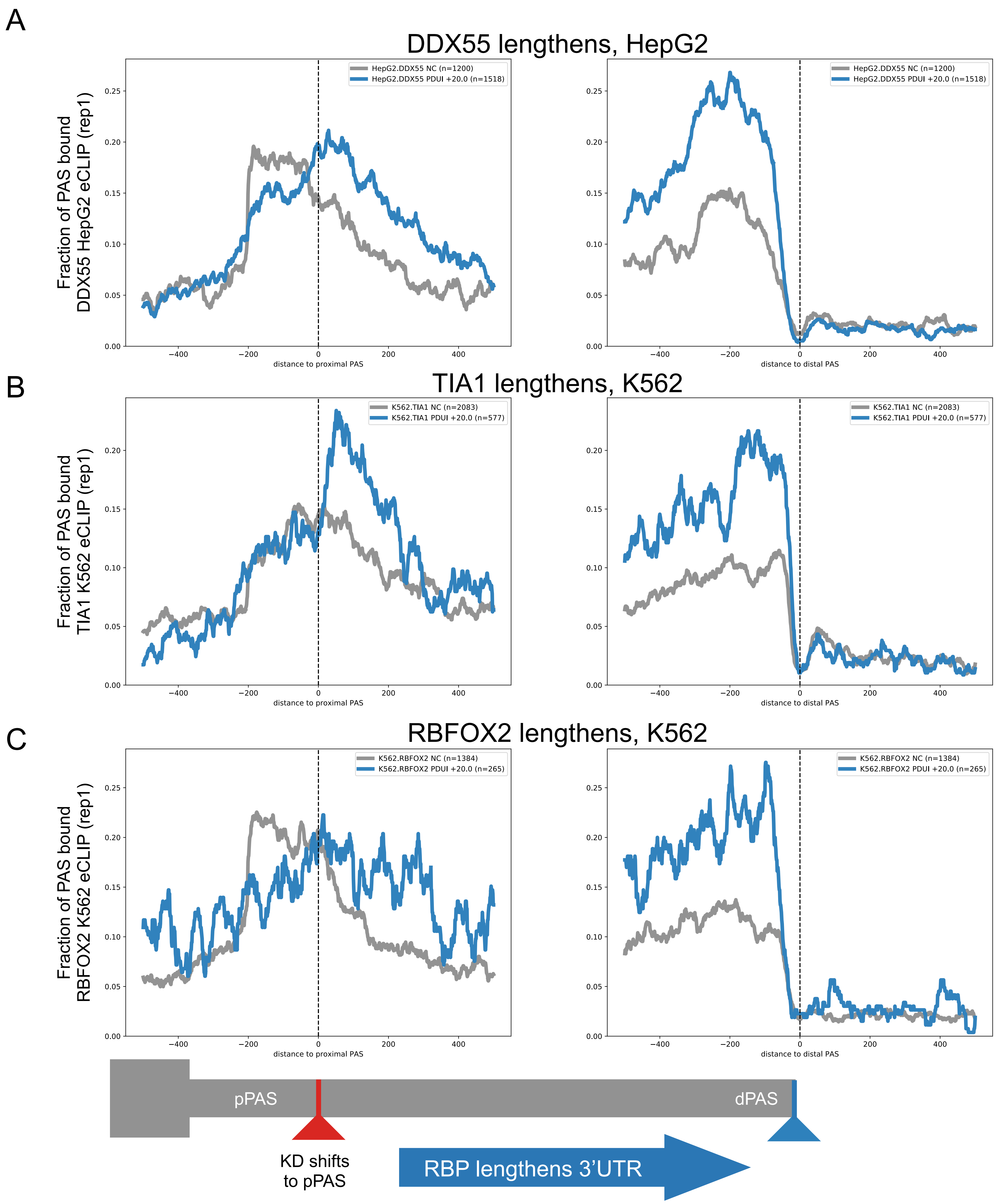

Fig. S8: **RNAmaps for putative direct regulators of promoting long 3'UTR isoform expression.**

(**A**) RNA map of the fraction of events with HepG2 DDX55 eCLIP peaks (replicate 1 shown) centered around proximal polyadenylation sites (pPAS, left) or distal PAS (dPAS, right) for events where DDX55 promotes 3'UTR long isoform expression (blue line, knockdown (KD) shifts expression towards pPAS, dDPUI≥20% with FDR<0.05) or events were DDX55 depletion had no effect on PAS choice (gray line, |dPDUI|≤5% with FDR>0.05). (**B**) As in (A), but for TIA1 knockdown RNA-seq and eCLIP in K562. (**C**) As in (A), but for RBFOX2 knockdown RNA-seq and eCLIP in K562.

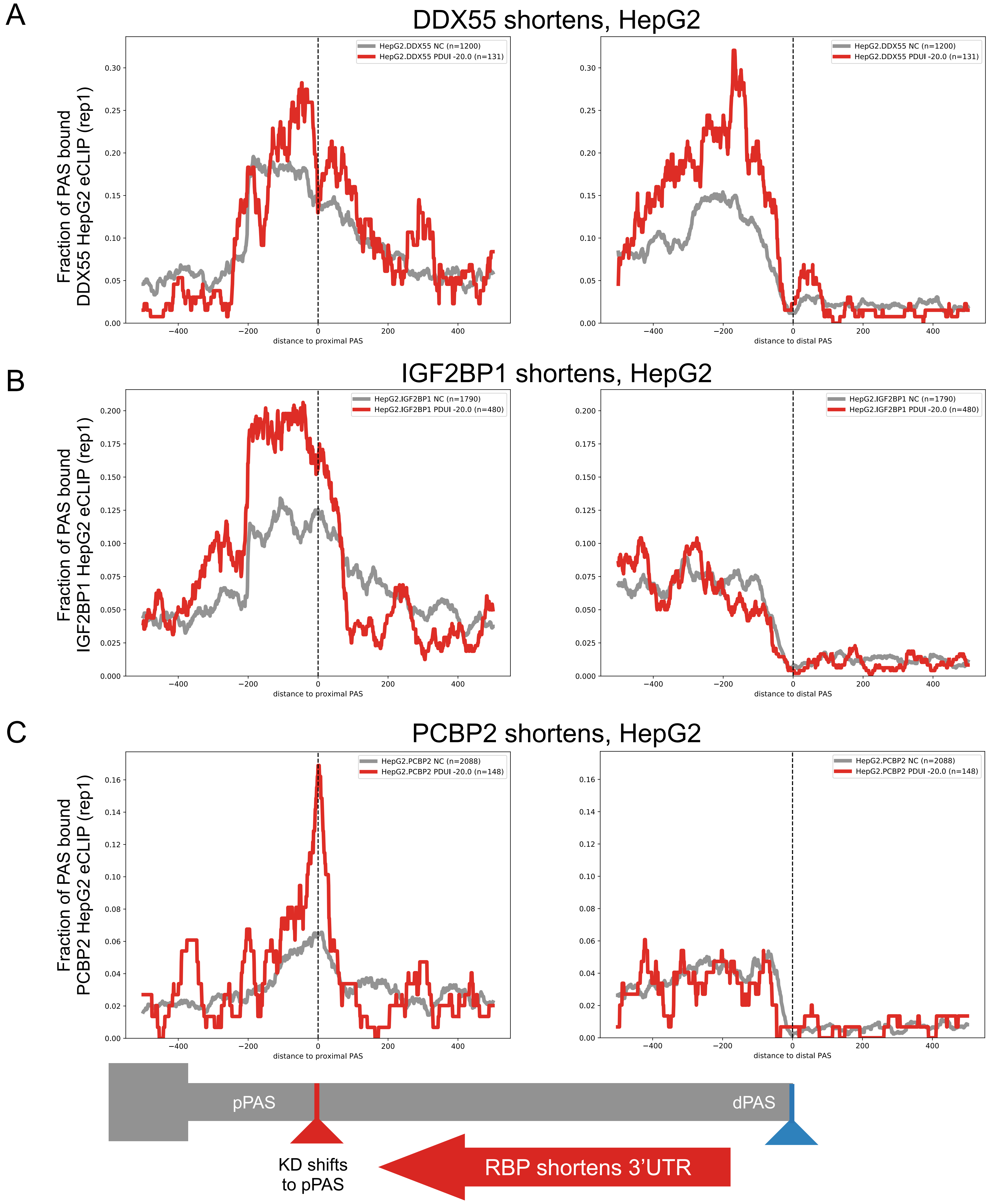

Fig. S9: **RNAmaps for putative direct regulators of promoting short 3'UTR isoform expression**

(**A**) RNA map of the fraction of events with HepG2 DDX55 eCLIP peaks (replicate 1 shown) centered around proximal polyadenylation sites (pPAS, left) or distal PAS (dPAS, right) for events where DDX55 promotes 3'UTR short isoform expression (red line, knockdown (KD) shifts expression towards dPAS, dDPUI≤-20% with FDR<0.05) or events were DDX55 depletion had no effect on PAS choice (gray line, |dPDUI|≤5% with FDR>0.05). (**B**) As in (A), but for IGF2BP1 knockdown RNA-seq and eCLIP in HepG2. (**C**) As in (A), but for PCBP2 knockdown RNA-seq and eCLIP in HepG2.

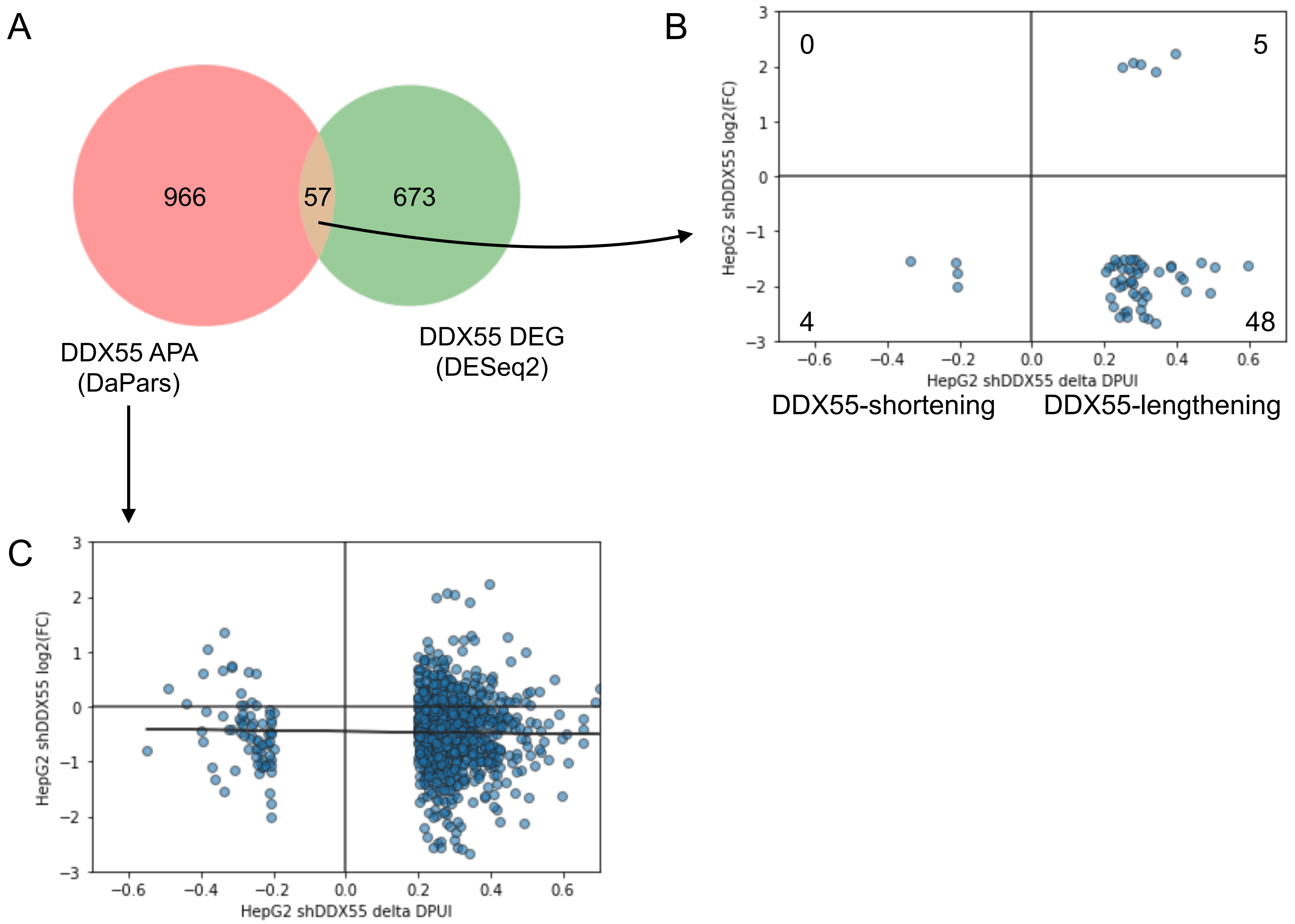

Fig. S10: **A subset of DDX55-dependent lengthening genes show decreased steady-state mRNA expression**

(A) Venn diagram of genes showing significant APA shifts in HepG2 cells upon DDX55 knockdown by DaPars (red) and differential expressed genes by DESeq2 (green). **(B)** Scatterplot showing the correlation between APA shifts (delta DPUI) and differential expression changes log2(fold-change) for the genes significantly called as both. **(C)** As in (B) but for all significant APA gene calls.

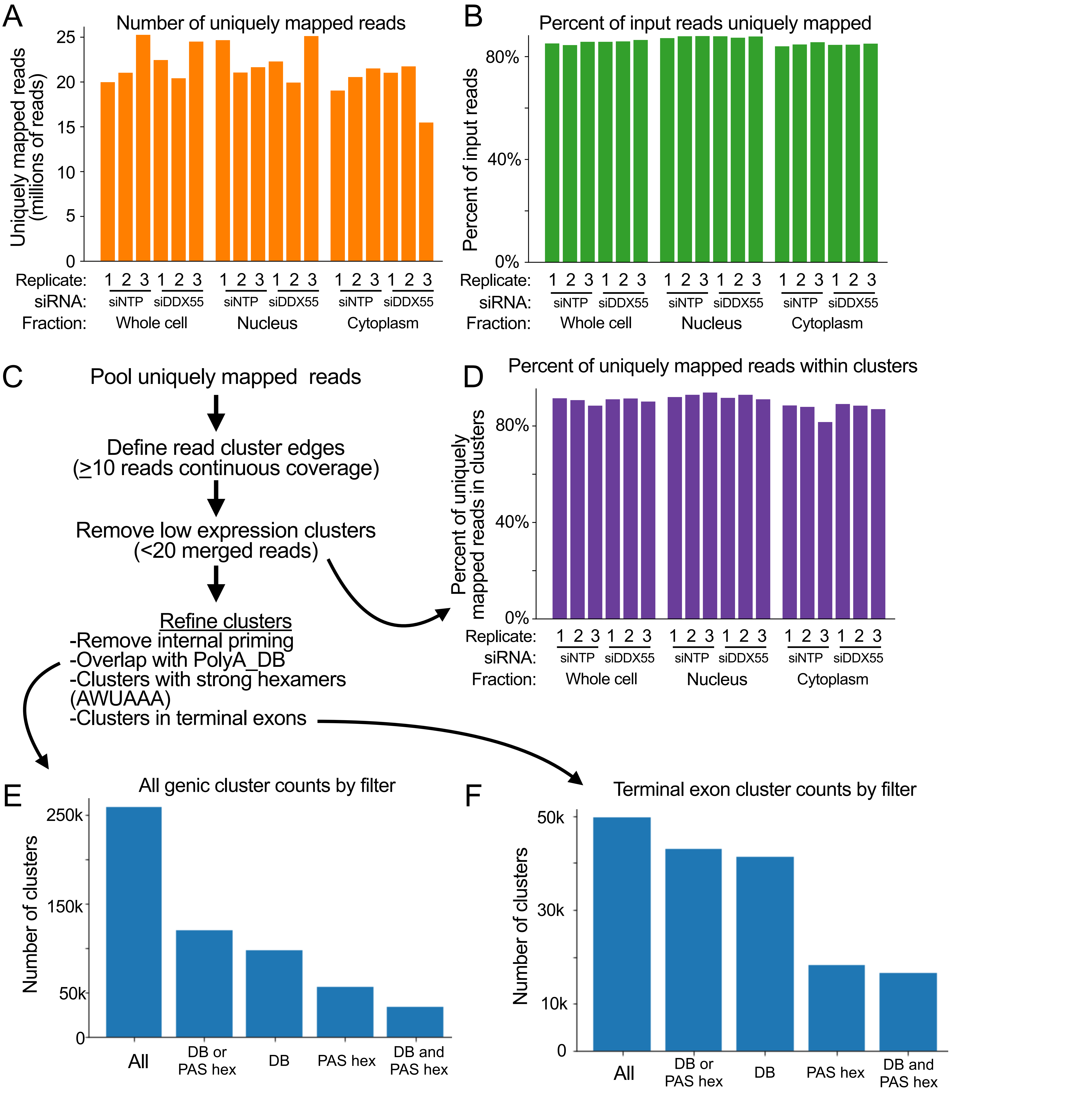

Fig. S11: **QuantSeq Rev V2 targeted 3'end sequencing summary statistics and QC**

(A) Number of uniquely mapped reads in millions per 3'end sequencing experiment as indicated. **(B)** Percent of input reads that were uniquely mapped to the genome per experiment. **(C)** 3'end cluster definition and refinement before calling relative PAS usage within each terminal exon. **(D)** Percent of uniquely mapped reads per experiment that fall within cluster boundaries **(E)** Number of distinct clusters that fall within or just downstream of gene regions (Ensembl v105) at with different levels of filtering as labeled. Filters include requiring overlap with PolyA_DB4 3'READ based sites (DB) and/or requiring overlap with the top two PAS hexamers (AWUAAA). **(F)** As in (E), but for clusters that overlap a terminal exon (Ensembl v105) that are the basis for calculating relative PAS usage.

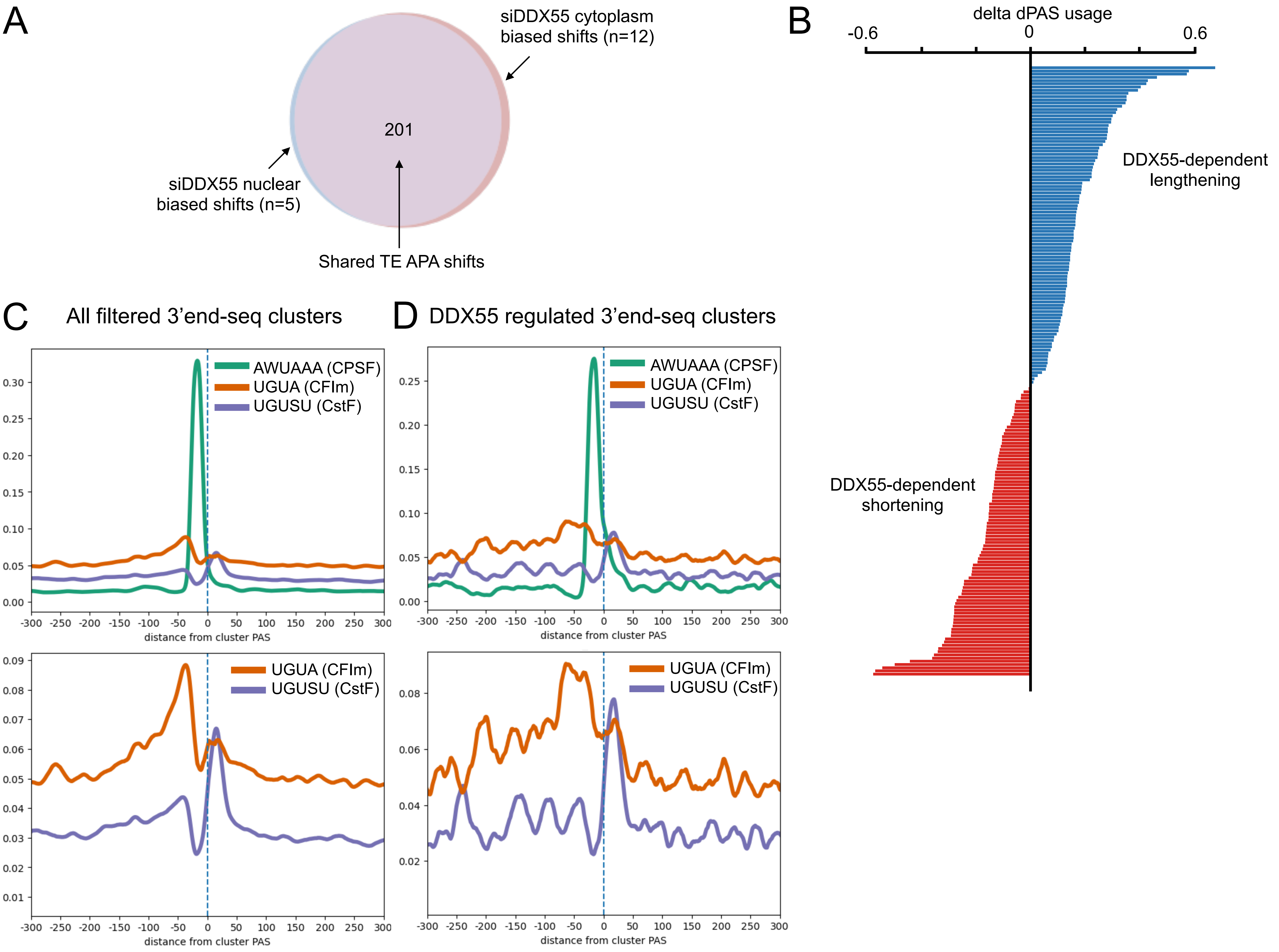

Fig. S12: **DDX55 terminal exons with tandem APA are largely shared between nuclear and cytoplasmic fractions**

(A) Overlap in high confidence terminal exons based on two PAS clusters with consistent change across two of three replicates in the nuclear or cytoplasmic fraction. Only terminal exons quantified in both compartments were considered **(B)** Barchart showing degree of change in distal PAS usage between control and siDDX55 knockdown in the cytoplasmic fraction for subset of shared TE APA shift exons. Blue indicates DDX55-dependent lengthening and red bars indicate DDX55-dependent shortening. **(C)** Motif map showing the per position occurrence of the core CPA motifs indicated over all filtered 3'end clusters. Motifs were counted in windows of 10 nts and a running mean of 10 nt was used to smooth the data. (**D)** As in (C) but for the subset of clusters regulated by DDX55.

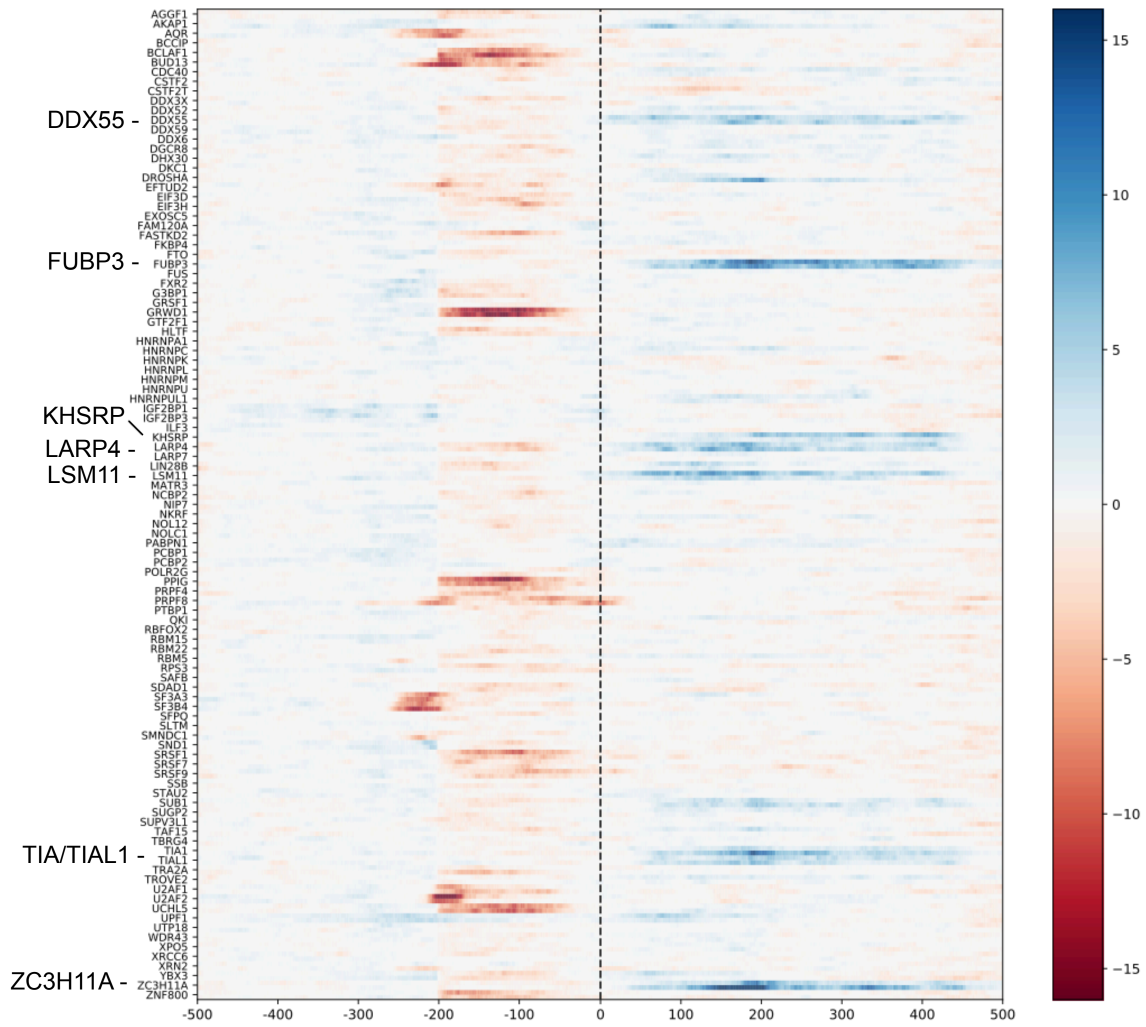

pPAS regulated by DDX55

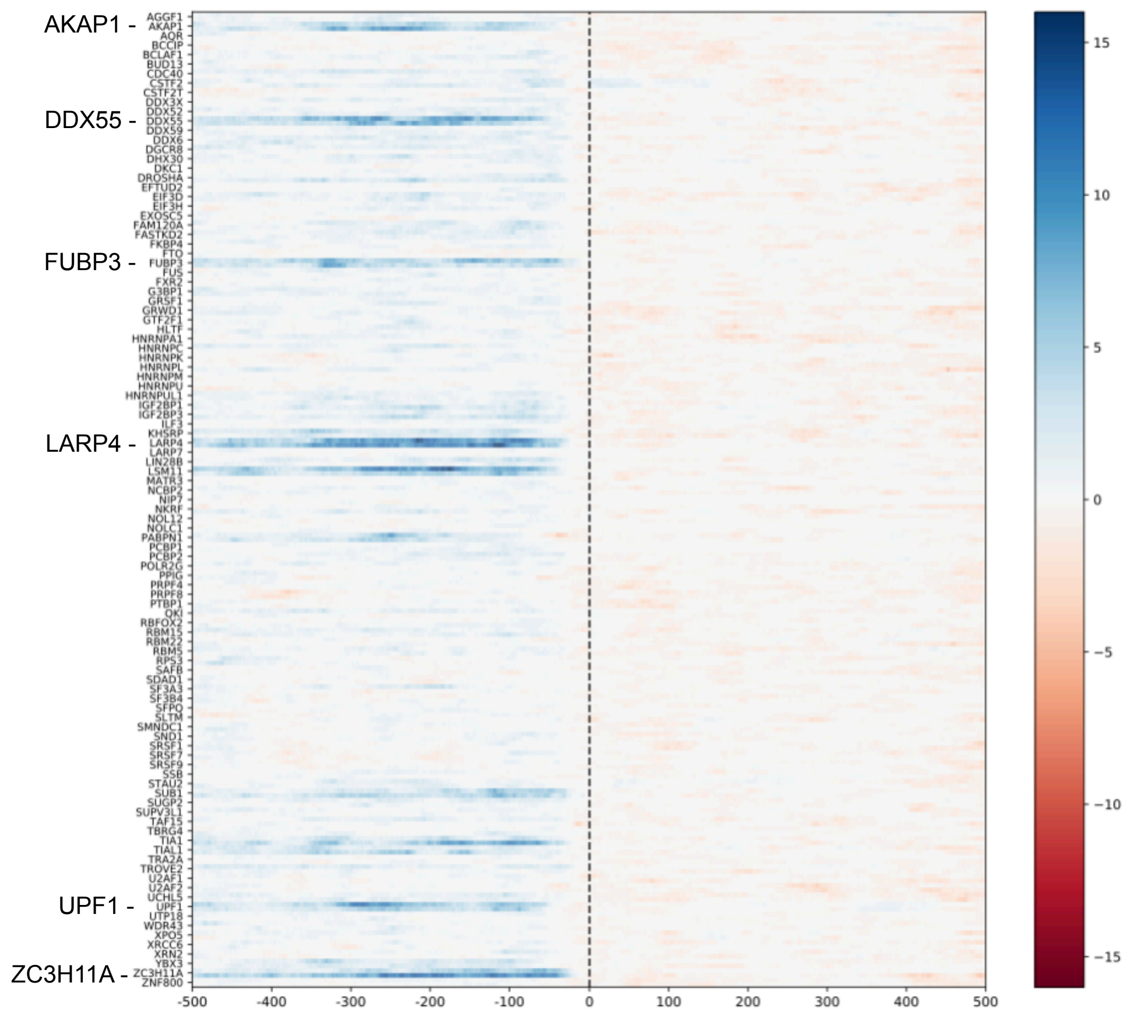

dPAS regulated by DDX55

Fig. S13: Correlation of other RBP eCLIP with DDX55 APA patterns

-log10(p-values) for enrichment or depletion of RBP eCLIP cluster occurrence between DDX55-dependent lengthened 3’UTRs versus non-changing 3’UTRs centered on the proximal PAS (Top) or distal PAS (Bottom).

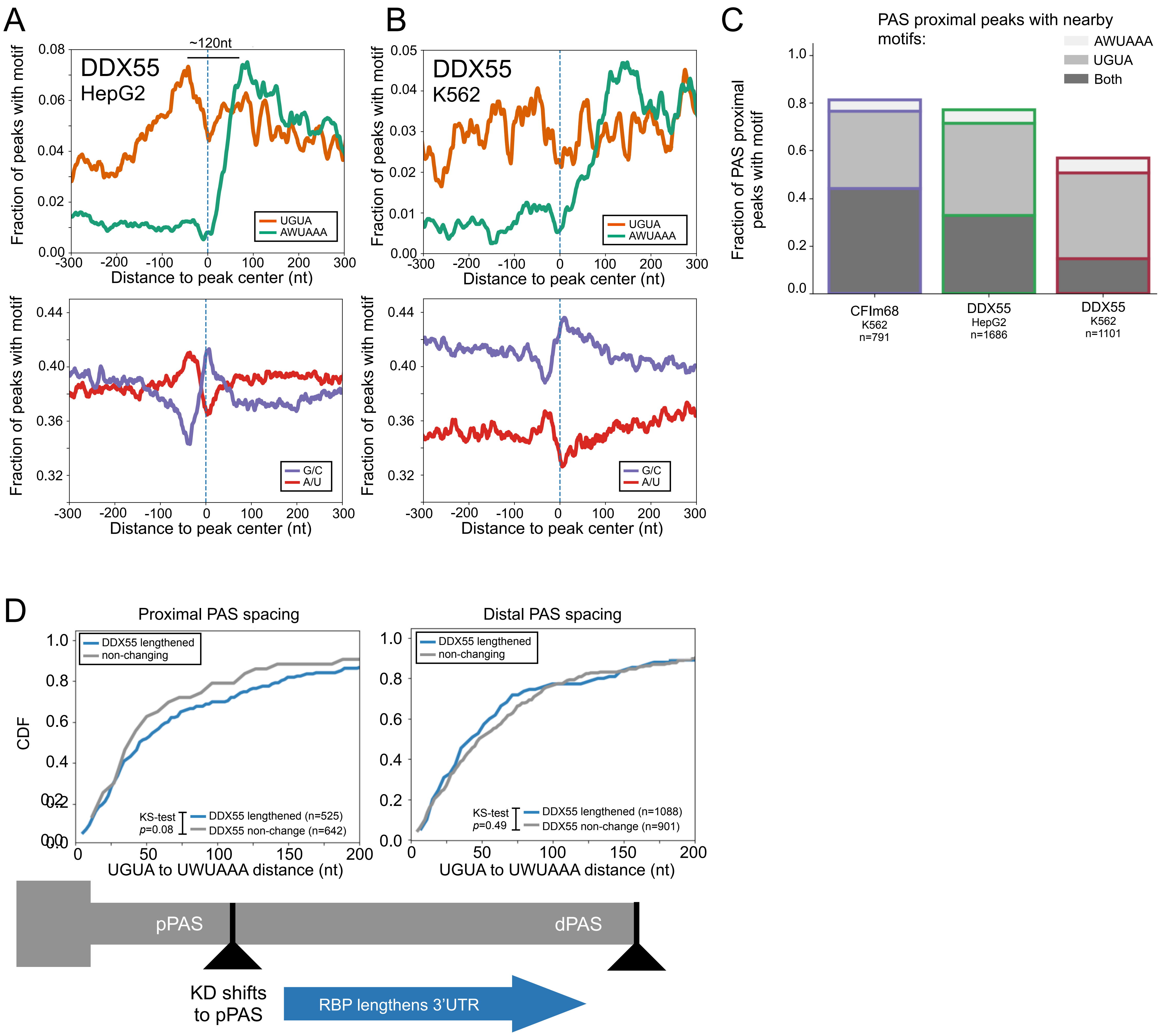

Fig. S14: Additional sequence features of DDX55 and CFIm binding.

(**A**) Motif maps showing per nucleotide frequency of the core PAS hexamers (AWUAAA, green) and CFIm upstream enhancing element (UGUA, orange) centered on the high confidence eCLIP peaks for DDX55 in HepG2 cells (top). Bottom motif map shows frequency of A/U (red) or G/C (purple) nucleotides over the same region centered on DDX55 peaks. Smoothing over a 10 nt window was applied. (**B**) As in (A), but for DDX55 high confidence eCLIP peaks in K562 cells. (**C**) Stacked bar chart showing fraction of subset of high confidence peaks for CFIm68 (CPSF6) or DDX55 near experimentally determined PAS which had one of the top two PAS hexamers alone (AWUAAA, light gray), the CFIm motif alone (UGUA, medium gray), or both (dark gray) within 150 nt of peak centers. (**D**) CDF of UGUA to AWUAAA motif distance occurring proximal to pPAS (left) and dPAS (right) that are regulated by DDX55 (blue, promotes 3'UTR lengthening) or non-regulated (gray).

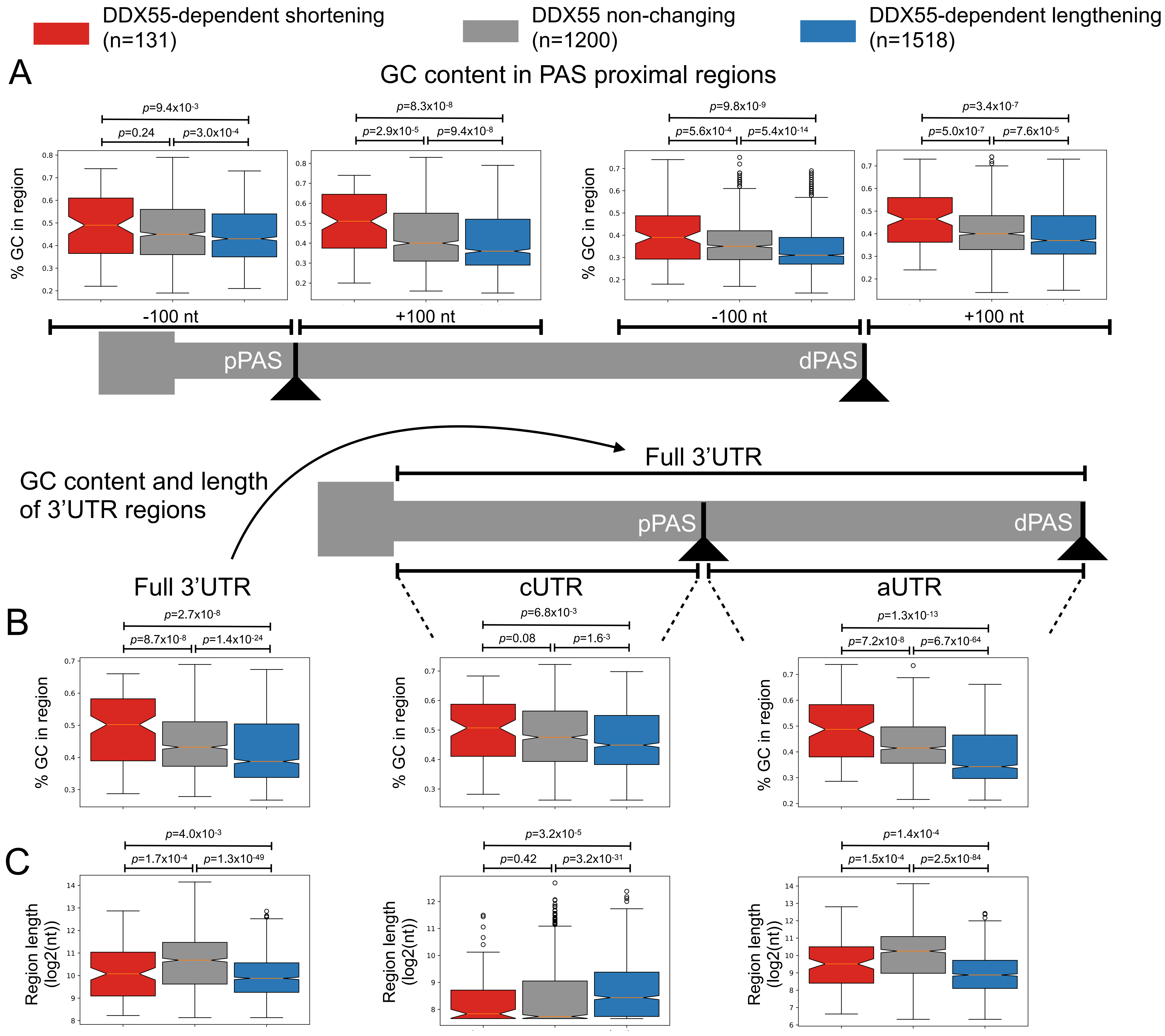

Fig. S15: **GC content in regions proximal to DDX55 regulated and non-regulated tandem PAS**

(**A**) Boxplots showing distribution of GC content in indicated regions within 100 nt upstream or downstream of proximal (pPAS) or distal (dPAS) sets of APA events indicated in the legend. Two-tailed KS test p-value is indicated for pairwise comparisons. **(B)** Boxplots for GC content for APA event types (statistics as in (A)) for indicated terminal exon regions: The full terminal exon (Full 3'UTR), the constitutive region shared between the short and long isoforms (cUTR), or the alternative region unique to the long terminal exon isoform (aUTR). Two-tailed KS test p-value is indicated for pairwise comparisons. **(C)** Boxplots showing distribution of each 3'UTR region length for the given set.

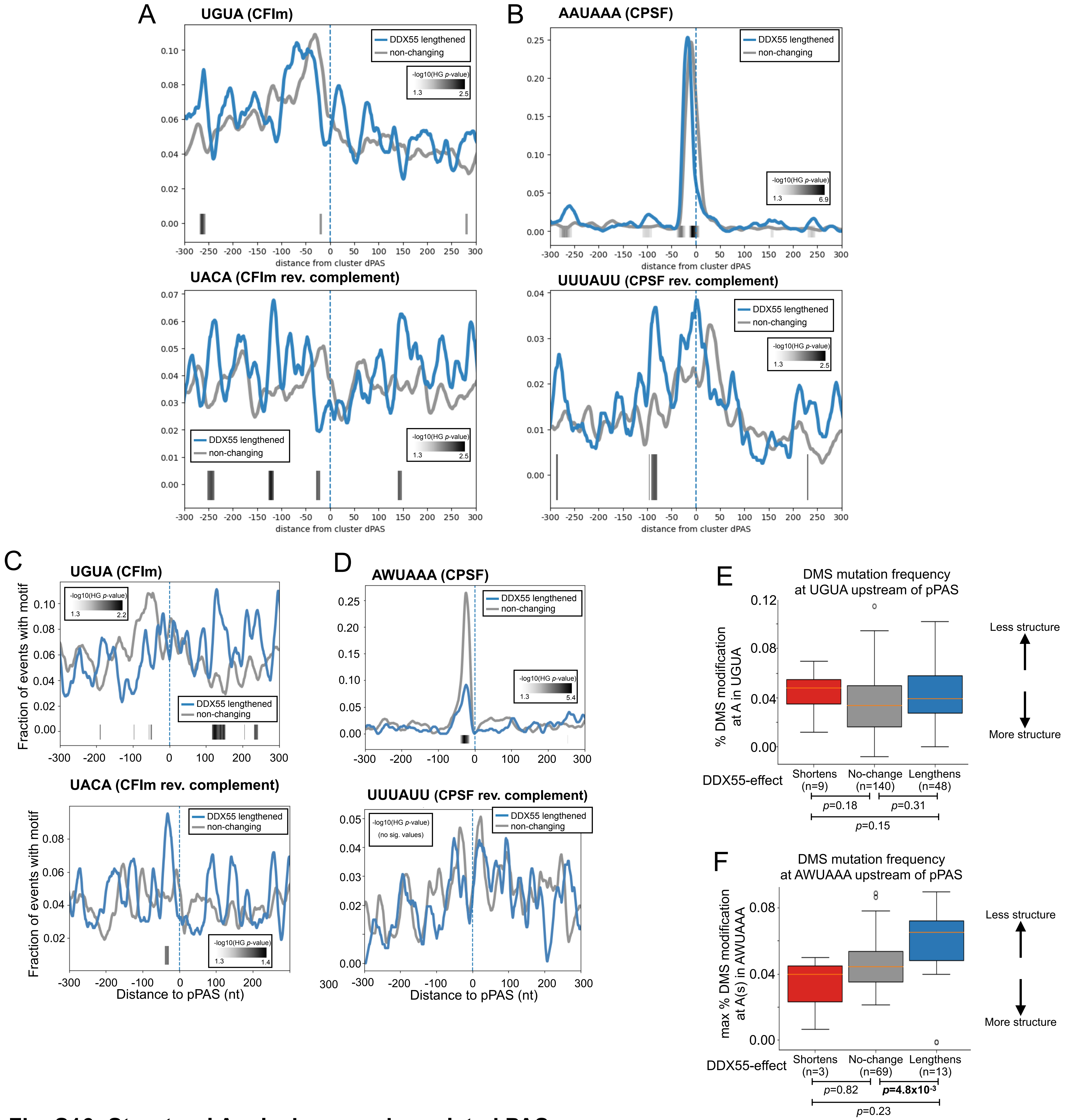

Fig. S16: Structural Analysis around regulated PAS

(**A**) Motif for CFIm and reverse complements around DDX55-dependent lengthened terminal exons determined by 3'end-sequencing data. Grayscale on the bottom indicates -log10(*p*-value) for positions of significant difference between positive and negative sets according to a two-tailed hypergeometric test. (**B**) Motif for CPSF and reverse complements around DDX55-dependent lengthened terminal exons determined by 3'end-sequencing data. (**C**) Motif maps showing per nucleotide frequencies of the CFIm binding site (UGUA, top) or its reverse complement (UACA, bottom) around proximal PAS (pPAS) regulated by DDX55 (blue) or non-changing (gray). Grayscale on the bottom indicates -log10(*p*-value) for positions of significant difference between positive and negative sets according to a two-tailed hypergeometric test. (**D**) As in (C), but for CPSF PAS hexamer (AAUAAA, top) or its reverse complement (UUUAUU, bottom) around proximal PAS. **(E)** Boxplot showing distribution of DMS modification rate for A nucleotides in UGUA motifs within 100 nt upstream of proximal PAS with sufficient data from DIM-2P-seq RNA accessibility dataset (Wu & Bartel 2017) (see Methods). **(F)** Boxplot showing distribution of maximum DMS modification rate for any A nucleotide in PAS hexamer motifs (AAUAAA or AUUAAA) within 100 nt upstream of proximal PAS with sufficient data from DIM-2P-seq RNA accessibility dataset (Wu & Bartel 2017) (see Methods). Two-tailed KS-test *p*-values are shown.

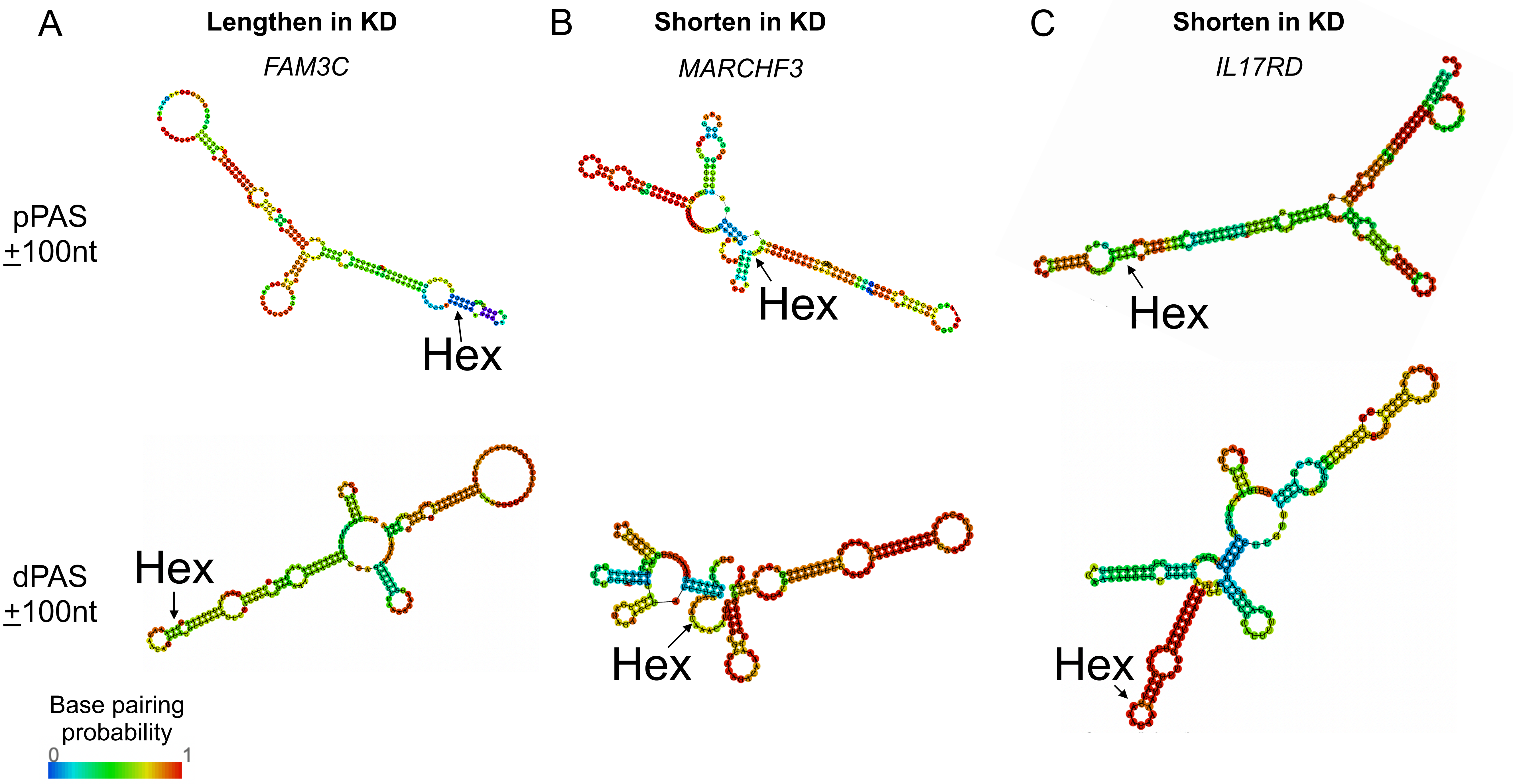

Fig. S17: *In silico* folding of 3'RACE validated DDX55 regulated APA events.

(A) RNAfold MFE secondary structure predictions for the region of 100 nt upstream and 100 nt downstream of the pPAS (top) or the dPAS (bottom) for *FAM3C*. The PAS hexamer (AAUAAA or AUUAAA) is labeled. Color scale indicates probability of base being unpaired where 1 is more likely unpaired. **(B)** As in (A) but for *MARCHF3.* **(C)** As in (A) but for *IL17RD*.
